# Using a human-centered, mixed methods approach to understand the patient waiting experience and its impact on medically underserved populations

**DOI:** 10.1186/s12913-022-08792-8

**Published:** 2022-11-22

**Authors:** Elizabeth N. Liao, Lara Z. Chehab, Kathryn Neville, Jennifer Liao, Devika Patel, Amanda Sammann

**Affiliations:** 1 Division of General Surgery, Department of General Surgery, University of California, 513 Parnassus Avenue, CA 94115 San Francisco, USA; 2 Department of Engineering Design, Stanford University, Stanford, USA; 3 Department of Emergency Medicine, Thomas Jefferson University Hospitals, Philadelphia, USA

**Keywords:** Waiting room, Patient experience, Medically underserved population, Outpatient clinic

## Abstract

**Purpose:**

To use a mixed methods approach to investigate the patient waiting experience for a medically underserved population at an outpatient surgical clinic.

**Methods:**

We used lean methodology to perform 96 time-tracked observations of the patient journey in clinic, documenting the duration of activities from arrival to departure. We also used human-centered design (HCD) to perform and analyze 43 semi-structured interviews to understand patients’ unmet needs.

**Results:**

Patients spent an average of 68.5% of their total clinic visit waiting to be seen. While the average visit was 95.8 minutes, over a quarter of visits (27%) were over 2 hours. Patients waited an average of 24.4 minutes in the waiting room and 41.2 minutes in the exam room; and only spent 19.7% of their visit with an attending provider and 11.8% with a medical assistant. Interviews revealed that patients arrive to their visit already frustrated due to difficulties related to scheduling and attending their appointment. This is exacerbated during the visit due to long wait times, perceived information opacity, and an uncomfortable waiting room, resulting in frustration and anxiety.

**Conclusions:**

While time tracking demonstrated that patients spend a majority of their visit waiting to be seen, HCD revealed that patient frustrations span the waiting experience from accessing the appointment to visit completion. These combined findings are crucial for intervention design and implementation for medically underserved populations to improve the quality and experience with healthcare and also address system inefficiencies such as long wait times.

## Introduction

Patient wait times are an important indicator of healthcare service delivery, as long wait times have been shown to negatively impact access to care and patient satisfaction [1–7]. In particular, patients from medically underserved populations not only have a higher burden of disease but also experience longer wait times [1, 8, 9]. For instance, Medicaid patients were found in one study to be 20 % more likely to wait longer than 20 minutes than their privately insured counterparts for an outpatient visit [10]. These factors further exacerbate their medical conditions and outcomes.

While the consequences of long wait times are well-described, patient perceptions of their waiting experience are poorly understood [11]. Several studies have attempted to characterize factors that affect the waiting experience, including communication, information transparency, trust and being respected [2, 11–13]. However, the number of aspects identified as affecting the waiting experience ranges from 11 in an emergency department [12] to 20 in a cancer radiology center [11]. Hospitals, providers, and payors use quantitative tools, such as the CAHPS Clinician and Group Survey (CG-CAHPS) [14], to assess the patient experience and quality of healthcare delivery, which fail to fully capture the multitude of factors that affect these experiences [15–17]. Similarly, qualitative studies cannot accurately take into consideration certain quantifiable patient experiences such as length of visit [13, 18, 19]. Of the few studies that used both quantitative and qualitative methods to describe the waiting experience, none have measured the waiting experience for patients from medically underserved populations [11, 20].

The goal of this study was therefore to apply a mixed methods approach to investigate the waiting experience for medically underserved patients at an urban safety-net hospital. By combining human-centered design (HCD) [21] and the lean methodology [22], we aimed to develop a deeper understanding of the current state of patients’ unmet needs at an elective surgery outpatient clinic.

## Methods

### Study design

**Fig. 1:**
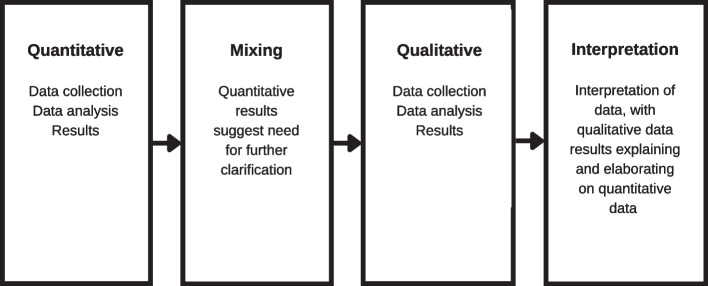
Prospective observational study design using a mixed methods approach

This study follows a prospective observational study design using a mixed methods approach (Fig. 1). Mixed methods approaches have been shown to provide a more accurate view of certain research topics [23], allowing us to uncover more multidimensional insights surrounding the patient experience. As such, we combined quantitative and qualitative data in order to better understand patients’ expressed and unexpressed needs in clinic. First, we collected quantitative data (February–May 2018) through retrospective chart review and components of the lean methodology to identify idle waiting times [24]. The lean methodology is an improvement process adapted from the car manufacturing industry and that has been broadly incorporated into healthcare quality improvement [22]. Then, to bring context to and build upon the quantitative findings, we subsequently collected qualitative data through HCD interviews (April–June 2018; June–July 2019.) HCD is an approach to problem-solving that relies on ethnographic research to understand unique challenges and unmet needs of stakeholders [21]. HCD research uses in-depth interviews and in-context observations to understand stakeholder needs, contexts, behaviors, and emotions. We used this dual approach due to the complementary nature of the two methodologies: while lean aims to identify and streamline inefficiencies in an established system, HCD aims to investigate how to redesign the system based on user-centered insights.

### Setting and population

This study was conducted at the Zuckerberg San Francisco General Hospital and Trauma Center (ZSFG), an urban safety-net hospital and level 1 trauma center in the city of San Francisco. ZSFG is an academic teaching hospital in the University of California, San Francisco (UCSF) network. The hospital treats medically underserved patients, including those who are indigent, un-insured or underinsured, are racial and ethnic minorities, and disenfranchised. The U.S. Department of Health and Human Services has defined such populations to be communities with members who have experienced health disparities, including refugees, religious minorities, and those identifying as African American [25]. As of 2021, almost all of ZSFG patients (96%) were publicly-insured (57% through Medi-Cal and 35% through Medicare.) Only 2% of patients were uninsured, while 4% of patients received care through private insurance [26]. The majority of patients were Hispanic (37%), followed by Asian/Pacific Islander (14%), White (17%) and Black (14%). Most patients were between 18 and 64 (71%), while 18% were over 64 years of age and 11% were under 18 years of age [26].

All study activities occurred at the general surgery outpatient clinic, which shares the same space with other surgical clinics, including podiatry, colorectal surgery, breast surgery, plastic surgery, and vascular surgery. At any given time, up to three services share the same clinical and waiting space. At the time of this study, the general surgery clinic was staffed by an attending general surgeon, a nurse practitioner (NP) and two medical assistants (MEAs). As an academic teaching hospital, medical students attend the clinic irregularly, depending on didactic and inpatient clinical activities. There are no residents staffed in the clinic because their rotation at ZSFG is in trauma surgery, rather than general surgery. Morning clinics were held Monday through Friday between 9 am and 12 pm, and afternoon clinics were held between 1 pm and 4 pm. There were 4 scheduled attending-led clinics per week (3 morning and 1 afternoon) and 1 scheduled NP-led clinic per week (afternoon.) This study was approved and informed consent was granted by the UCSF Institutional Review Board.

### Quantitative data collection and analysis

Patient age, gender, ethnicity, race, primary home language, family size, and income source were collected and analyzed in February 2018 via chart review on patients who had a scheduled patient visit at the general surgery outpatient clinic from Jan 1, 2008 to Jan 1, 2018. We conducted this analysis to understand the sociodemographic distribution of our patients.

Using the lean methodology, three quantitative researchers tracked patients throughout a clinic visit, documenting times for the following activities: patient entered the waiting room; patient entered the exam room; MEA entry and exit from the exam room; attending surgeon or NP entry and exit from the exam room; patient exited the exam room; patient exited clinic. Researchers observed 11 general surgery clinics between February 2018 and May 2018 and tracked all patients who attended their appointment during these clinics.

Data were collected in a custom-built Microsoft Excel 2011 tool that used a circular formula to timestamp the start and end of each activity to track duration and frequency. Quantitative researchers were trained in the use of this tool and educated on how to identify and code each activity. Data were analyzed using basic frequency and descriptive statistics for the following activities: time patient spent waiting (in the waiting room and exam room); time spent with the provider (surgeon and/or NP); and time with staff (time with MEA taking vitals and/or additional visits).

### Qualitative data collection and analysis

We used HCD to conduct and analyze semi-structured interviews with patients about their experience in clinic. HCD provides a unique approach for homing in on problems and finding solutions for them. As such, during the interview process and when analyzing the interviews, we focus on understanding the challenges and unmet needs of each stakeholder. This differs from traditional qualitative interview techniques, which focus on observing a culture, understanding an experience, and developing a theory [27].

Three qualitative researchers trained in HCD interview methods conducted “intercept” interviews with a convenience sample of patients during two time periods: April to June 2018, and June to July 2019. Intercept interviews are conducted while the participant is still on site and engaged with the experience or product in question. They are commonly used in consumer research where potential participants are difficult to reach and engagement with the experience or product is key to the interview [28, 29]. These researchers were different than the quantitative researchers who performed time-tracking. All adult, English-speaking patients who were in the waiting room at the time of interviews were eligible for inclusion. Patients were asked if they would like to participate and if they gave verbal consent, the interview commenced immediately, allowing for real-time reactions to situations and environments. The interview concluded when the patient or researcher ended the interview, or when the patient was called by staff to exit the waiting room. Interviews lasted under 30 minutes and aimed to elicit perspectives on patients’ experiences with the surgical clinic, focusing on waiting periods. Interviews were anonymous to preserve patient privacy and initials were randomly generated for each patient. Notes and key quotes were documented during the interview, and recruitment ended once thematic saturation was reached.

**Fig. 2:**
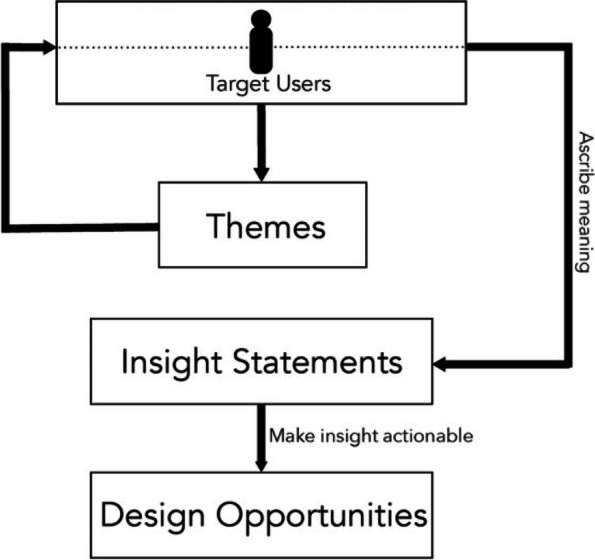
Design process of developing insights. Figure taken from one of our previous papers, Nijagal MA et al. [33]

Two of the qualitative researchers independently performed inductive analysis in order to identify initial themes, in accordance with the HCD method for qualitative analysis [30]. They met to develop and refine themes, consolidate based on redundancy, and group them into thematic categories. Discrepancies were reconciled through discussion. Key quotes associated with each thematic category were extracted from interview notes, after which the researchers met to ensure that supporting quotes and descriptions for each thematic category were defined and agreed upon. Per the HCD methodology, the researchers then extrapolated ‘insight statements’ from these themes [31]. Insight statement development is an integral step in the HCD analysis process and involves re-reviewing the notes to understand themes in the context of the individual interviews in order to deduce unique human perspectives, motivations, or tensions from the thematic data [31, 32] (See Fig. 2 for design process of developing insights).

## Results

### Study population

17,632 patients scheduled 65,211 visits with the general surgery outpatient clinic from 2008 to 2018. Their sociodemographic characteristics are described in Table 1. This population was considerably diverse: the average age of the patients at the visit was 52.5 years (SD 15.3), 11,412 (64.7%) patients identified as male, 13,097 (74.2%) did not identify as Non-Hispanic White, and 5937 (33.6) did not speak English as their primary language. 14,305 (81.1%) were the only family members in their household and 10,767 (61.1%) listed that they had no income source.

**Table 1:** Sociodemographics of patients seen in general surgery outpatient clinic from 2008 to 2018

Demographics	Patients seen (*n* = 17,632) No. (%)
Age, average (standard deviation), years	52.5 (15.3)
Gender	
Female	
Male	11,412 (64.7)
Race/Ethnicity	
Hispanic	1153 (6.5)
Non-Hispanic Black	3053 (17.3)
Non-Hispanic Asian	3285 (18.6)
Non-Hispanic White	4535 (25.7)
Non-Hispanic Other	5280 (30.0)
Unknown or Decline to States	326 (1.8)
Primary language	
English	11,695 (66.3)
Spanish	3303 (18.7)
Cantonese	1320 (7.5)
Other language	1166 (6.6)
Unknown	148 (0.84)
Family size	
One member	14,305 (81.1)
Two members	1807 (10.3)
Three members	678 (3.9)
Four members	521 (3.0)
Greater than four members	321 (1.8)
Income source	
Professional/technical^a^	343 (2.0)
Labor/production^b^	1144 (6.5)
Service/sales	2148 (12.2)
Retirement income	336 (1.9)
Disability income	822 (4.7)
General or public assistance	1023 (5.8)
Other^c^	1049 (6.0)
None	10,767 (61.1)

^a^This is inclusive of executive, administrative, managerial, professional, technical and related support)

^b^This is inclusive of production, inspection, repair, craft, handlers, helpers, labors, and transportation

^c^This is inclusive of Veteran Affairs benefits, interest, dividends, rent, child support, alimony, etc.

### Quantitative time tracking

**Fig. 3:**
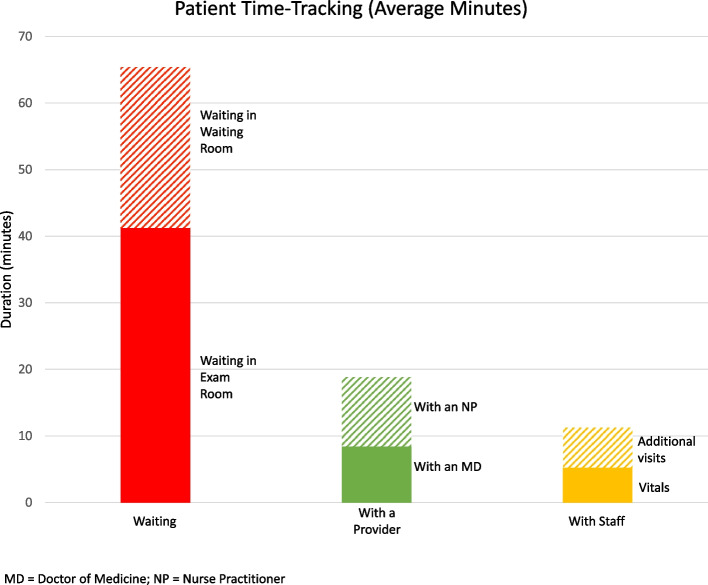
Patient Time-Tracking Results

We documented the patient journey for 96 patients across 11 clinics, each led by 5 attending surgeons (Table 2). The average patient visit lasted 95.8 minutes. About a quarter of the visits (*n* = 26, 27%) were over 2 hours long. On average, patients spent 68.5% of their visit waiting to be seen by a provider or staff member. While a majority of their waiting period was spent alone in their exam room (43.0%), the remainder was spent in the clinic waiting room (25.5%). Patients spent 19.7% of their visit with the attending physician or a nurse practitioner, and 11.8% of their visit with a staff member. Of the time spent with a staff member, 5.4% of the visit was spent performing vitals and 6.6% of the visit was spent on additional visits with an MEA (Fig. 3).

**Table 2:** Patient time-tracking results

Patient Time Tracking	Average Duration (mins)	Minimum Duration (mins)	Maximum Duration (mins)
Waiting to be seen	65.6	0.2	232.9
Waiting in exam room	41.2	0.2	122.9
Waiting in waiting room	24.4	0.0	110
With an MD and/or NP	18.9	1.0	53.3
MD	8.4	0.7	27.0
NP	10.5	0.3	26.3
Time spent with other staff	11.3	1.6	37.2
Vitals	5.2	1.5	14.3
Additional visits	6.1	0.1	22.9

*MD* Doctor of Medicine, *NP* Nurse Practitioner

### Insights from qualitative interviews

Analysis of 43 interviews with patients and their families revealed 6 distinct insights: 3 were related to patients’ “pre-visit experience”, and 3 were related to the “during visit experience” (Table 3).

**Table 3:** Representative patient quotes for each insight

Insight	Representative patient quotes
Patients’ schedules are not prioritized when making an appointment in clinic, leading to emotional distress and repeated non-attendance.	● Patients’ schedules are not prioritized when making an appointment in clinic... ○ “Maybe it’s MediCal but it took me a long time (1.5 years) to get surgery” (AN) ○ “I can’t schedule any appts around meals bc of my client” (AY) ○ “I called 6x to get this appt, call two different places -- struggled to get appt..I was told I’d get a call from 3 M to remove stitches and to call the clinic if I didn’t hear from them.” (BC) ○ “I had to fight to get this appointment to make a day that I could physically be in person to make this appointment” (AN) ○ “I try to make my doctors a priority but it’s hard when you have so many appts” (BD) ○ “I tried to call to reschedule my appointment, I couldn’t” (BF) ● ...leading to emotional distress and repeated non-attendance. ○ “my family already told me to sue because they’re playing with my health” (BH)
Patients struggle to find the support necessary to attend their appointment, leading to frequent non-attendance and increased socioeconomic stressors.	● Patients struggle to find the support necessary to attend their appointment… ○ “I’m retired, I take care of my grand babies, take my daughter to work…that’s why I have AM appts, so I can take everyone in the morning then come to my appt” (AQ) ○ “I’m the oldest in my family, I’m the person taking care of everything” (AZ) ○ “I’m a care provider through...my goal in life is to keep my clients alive and out of the hospital” (AY) ● ...leading to frequent non-attendance and increased socioeconomic stressors. ○ “My medical bills wipe me out...my friends keep telling me to move to Thailand or Germany, telling me it’s so much better here.” (AX) ○ “With housing, i feel more stable, I don’t worry about taking all my stuff with me to appointments” (BB)
Patients lack accessible transportation options that fit within their medical and socioeconomic constraints, leading to missed appointments and delays of care.	● Driving self ○ “I drove starting at 3 AM to get here today” (AL) ○ “I take the bus now...driving is challenging because of the parking…there’s only 1 hour parking around the hospital.” (AN) ○ “Once I had to wait for 45 minutes in a room, and got a $75 dollar parking ticket.” (BO) ● Getting a ride from friends and family ○ “My son drove me here and will pick me up. I have to be dropped off early in the morning before my son goes to work and wait for him to pick me up.” (BP) ● Calling a taxi or uber/lyft ○ “I gotta get better... I missed some appts...I try to make this a priority but every time I have to take a taxi over here, it’s expensive” (BD) ● Using public transport ○ “I like to get here ½ hr. before, today it was 10 min before because my bus was late and all the tech bus traffic.” (AY) ○ “I got lost from the bus stop, it’s a long walk, I had no idea where 3 M clinic was” (AV) ○ “I rely on the MUNI, I’ve been late a couple of times because of it.” (AX) ● Mobility issues ○ “[the clinic is] on the third floor. Even the check in place was pretty far from the elevator. All the way to this waiting room is not the shortest walk that I’ve done” (BJ) [has crutches] ● ...leading to missed appointments and delays of care ○ “They like us to be on time or else we miss our *appointment*” (BH)
Patients spend the majority of their visit in the clinic waiting to be seen by a provider, leading to anxiety and frustration.	● Patients spend the majority of their visit in the clinic waiting to be seen by a provider… ○ “You have to come half an hour and then wait another half hour or 45 minutes. I feel that’s too long” ○ “The procedure is not even 5 minutes but sometimes I have to sit in the room waiting for 45 minutes. I can’t do anything while I’m waiting.” (BN) ○ “Wait time is long but I can’t complain, there’s a reason why, but I’ve heard horror stories” (AG) ○ “The waits are too long..there’s a lot of patients overlapping in time” (BL) ● ...leading to anxiety and frustration. ○ “I’m here because it’s either be here or be in pain…I wouldn’t come if I had to wait this long and my leg didn’t hurt” (AU) ○ “Waiting is the worst part...but that’s just life” (BD) ○ “You wait a long time here...I got here early hoping they might be able to see me earlier” (AO) ○ “Lots of waiting here but I can’t complain...if you come earlier, they might see you earlier” (BE) ○ “The wait is too long…should be faster, I got things to do...what’s the point in scheduling if they’re always late? Sometimes I come late on purpose.” (AU)
Information opacity related to their clinic appointment makes patients feel disrespected and incapable of managing their medical conditions, leading to frustration and disempowerment.	● Information opacity related to their clinic appointment makes patients feel disrespected and incapable of managing their medical conditions… ○ “When I’ve come to clinic in the past, I’ve arrived only to find that my appt was cancelled or moved...they didn’t call me to notify me” (AY) ○ “I had to call beforehand to know where [the clinic] is. Otherwise, it was kinda unclear how to find the 3 M clinic.” (BJ) ○ “I called the clinic 6x, they didn’t pick up the phone, so I left a voicemail” (BB) ● ...leading to frustration and disempowerment. ○ “Is my doctor in? No? Well of course not. He is definitely going to be late.” (BM) ○ “I’ve been dealing with this hernia that I shouldn’t have... I don’t know if it was the method that was used, weakness in my stomach wall and that I overexerted...I followed all the directions but I still ended up injuring myself post surgery. I’m not happy with this, it was not explained, I wasn’t given adequate instruction to protect myself. (AK) ○ “Getting through to a person in clinic is challenging…patience is on my part to wait” (AK) ○ “I don’t like complaining…I’m so frustrated, I’m crying….What else can you do? I’m not going to go off on no body which I feel like doing, but I’m better than that. I have more patience… that’s all I can do. What else can you do?” (BH)
An uncomfortable physical clinical environment exacerbates patients’ anxieties related to waiting for their appointment and adversely impacts their mental and emotional states.	● An uncomfortable physical clinical environment exacerbates patients’ anxieties related to waiting for their appointment… ○ “Most of the people are dirty, filthy” (BL) ○ “it’s nice and it’s clean” (BG) ○ “[Waiting room is] not clean, there are so many types of people (AH) ○ “As long as it’s clean here [WR], I’m fine with it.” (AQ) ○ “Small room, gets pretty packed…I’d like a bigger WR or two.” (AC) ○ “First of all, it’s crowded.” (BJ) ○ “It’s a small waiting room. I don’t know if it gets packed this often, but maybe a larger waiting room closer to the elevators or something like that” (BJ, had crutches; in response to how to improve the waiting room) ○ “I like early morning appointments because it gets too crowded later” (AQ) ○ “when that man stands up, his seat will be wet” (BM) ● ...and adversely impacts their mental and emotional states. ○ “I cannot afford to have my conditions getting worse due to catching germs unnecessarily” (BL) ○ “I’m not hanging in there with this waiting room. I’m not doing too good at all” (BO) ○ “It needs something calming...not stuff that’s rousing of emotion” (AN) ● Preferred an environment that was calming ○ “[Emergency department] was much less crowded, quieter, we didn’t have the TV on that I don’t necessarily want to listen to” (BJ) ○ “We need one more central WR, people spend too much time walking around” (AX) ● Preferred an environment that included activities/amenities ○ “I liked that my other clinic has a computer” (AC) ○ “They should have a system where you they call you when you’re up next so you can step out and get a coffee” (AO) ○ “More comfortable chairs would nice...but you’re not going to be in here long” (AS) ○ “As long as I get care, this [WR] is fine. You can sit in places that have VR headsets but then you have to pay $1000” (AW) ○ “I’m on Medicare so I don’t feel like I have control of amenities” (AW)

#### Pre-visit experience

##### Insight 1

Patients’ schedules and personal constraints are not prioritized when making an appointment, making patients feel the need to “fight” to access their care.

*Description:*

For many patients, the waiting experience started with scheduling an appointment. Patients’ feedback around scheduling could be categorized into 3 issues: not being able to get an appointment in a timely manner, not being able to get an appointment at a time that accommodates their schedule, and last-minute cancellations from the clinic with minimal or no notification. These issues caused a significant amount of emotional distress for the patients. One patient (BH) expressed it as feeling like the doctors were playing with her health. Patients felt that they needed to “fight” (AN) and “sue” (BH) to be seen and taken care of by their providers. In addition to emotional distress felt by patients, patients also reported that difficulties in scheduling their appointment resulted in delays of care. For instance, it took AN “1.5 years” to get their surgery done. Finally, it was not an infrequent occurrence for patients to learn that their appointment was canceled or rescheduled only after they arrived in clinic. This exacerbated their sense of frustration and mistrust as they reported having already made the difficult rearrangements to attend their appointment.

##### Insight 2

Patients struggle to find the support necessary to attend their appointment, leading to frequent non-attendance and increased socioeconomic stressors.

*Description:*

The clinic operates during standard work hours, which conflicts with primary work responsibilities for many patients. Patients are low-income and have jobs and responsibilities for which they are unable to miss without significant consequences. As such, the opportunity cost of attending clinic appointments is high – it may mean reduced pay or leaving loved ones unattended. Many patients reported missing their appointments for these reasons. For instance, AY is a care provider and lives with her client “24/7”. Her goal in life “is to keep clients alive and out of the hospital”. Finding a time to make it to the appointment while also ensuring that her client is taken care of in her absence is difficult and rare.

##### Insight 3

Patients lack affordable and accessible transportation options that fit within their medical and socioeconomic constraints, leading to increased stress and missed appointments. *Description:*

Transportation was a common barrier to attendance. Patients reported using the following forms of transportation: driving their own cars, having other people give them a ride, taking a taxi or rideshare, and taking public transportation. Each had limitations; there was no ideal option. Due to the high cost of living within the Bay Area and in San Francisco, many of our patients live over 20 miles away from the hospital. This resulted in patients making significant rearrangements to make it to the clinic on time. “I drove starting at 3AM to get here today” (AL), was a typical experience echoed by many patients. For those that were late to their appointment or not able to make their appointment, patients were labeled as “no-show” and had their appointment canceled. This resulted in delays in care and poor patient experience.

For those who drove their own cars, many cited parking as a source of stress. At the time of our interviews, there was a 1-hour limit enforced on street parking surrounding the hospital. With the average visit lasting over an hour, and many lasting more than 2 hours, parking is a nontrivial matter. Deciding whether to step out of clinic to move their car or to stay and risk getting a hefty fine, added a significant amount of stress for patients. As BO stated: “Once I had to wait for 45 minutes in a room, and got a $75 dollar parking ticket.” Patients worried that they would miss their turn getting called for their visit if they left clinic, thereby increasing their waiting time to see their provider.

Patients without personal transportation either sacrifice time or money. For patients who relied on friends or family to drive them to their appointment, they had to sacrifice some of their own time to make the appointment. For instance, BP had to work around his son’s schedule, resulting in him being “dropped off early in the morning before [his] son goes to work and waiting for him to pick [BP] up.” Those who took taxis or ride shares found the cost prohibitive. As BD stated: “I missed some appointments...I try to make this a priority but every time I have to take a taxi over here, it’s expensive.” Those who took public transport sometimes got lost or were late to their appointments because the transportation was not reliable. As AV stated, “I got lost from the bus stop, it’s a long walk, I had no idea where [the clinic] was.”

Even once they reached the hospital, patients faced difficulty getting to the clinic. Many patients used a wheelchair or crutches or had other mobility issues; the clinic is on the third floor and is not close to the elevator. On top of that, patients frequently got lost in the hospital while looking for the clinic. As BJ, who used crutches, said: “[the clinic is] on the third floor. Even the check in place was pretty far from the elevator. All the way to this waiting room is not the shortest walk that I’ve done.”

#### During visit experience

##### Insight 4

Patients spend most of their visit in the clinic waiting to be seen by a provider, leading to anxiety and frustration.

*Description:*

Patients reported wanting to spend a meaningful amount of time with their care team, and for the time spent waiting to be outweighed by the benefits of being seen by a provider. However, many expressed doubts that this was the case, and wondered aloud if their visit justified the hardships they faced in accessing their appointment and the experience of waiting to be seen by their provider. As BN put it: “The procedure is not even five minutes but sometimes I have to sit in the room waiting for 45 minutes. I can’t do anything while I’m waiting.” Another patient (AU) put it this way: “I’m here because it’s either be here or be in pain…I wouldn’t come if I had to wait this long, and my leg didn’t hurt”.

Patients managed their frustration and anxiety with extended wait times in different ways. Some patients, like BE and AU, developed workarounds in attempt to decrease their waiting time, such as coming in earlier or later than their appointment time. Others felt resigned to waiting; they felt that it was a part of life or that things could be worse, so they chose not to complain about it. As AG stated, “wait time is long, but I can’t complain, there’s a reason why, but I’ve heard horror stories.”

##### Insight 5

Information opacity makes patients feel disrespected and incapable of managing their medical conditions, leading to frustration and disempowerment.

*Description:*

Patients wanted information that allowed them to participate in their care and sought information at two points in time: before their clinic appointment and during their visit. Unfortunately, there was information opacity at both points. For the former, the questions centered around logistics. As noted in insights 1 and 3, patients often did not receive enough information about their appointment or where the clinic was located. For the latter, patients wanted updated waiting time estimates and to learn how to manage their medical conditions. While patients were dissatisfied with the long wait times, they were especially frustrated that they did not know when they would be called – especially after their original appointment time had passed. In addition, some patients stated they experienced disease progression and complications because they did not receive adequate patient instruction on how to take care of themselves and their health conditions. As AK explained: “I followed all the directions, but I still ended up injuring myself post-surgery. I’m not happy with this, it was not explained, I wasn’t given adequate instruction to protect myself.”

Patients responded to this information opacity in a variety of ways. Most expressed feelings of frustration and disrespect. Some expressed resignation and felt that complaining wouldn’t lead to their desired outcome; all they could do was practice patience and endure. For instance, BH was waiting to be seen and was frustrated that her hernia repair had been delayed. Even though she had been waiting in the waiting room for a long time and was angry at the providers and hospital to the point of breaking down and crying during our interview, she decided to not “go off on no body which I feel like doing...I have more patience...that’s all I can do. What else can you do?”

##### Insight 6

An uncomfortable physical environment in clinic exacerbates patients’ anxieties related to waiting for their appointment, adversely impacting their mental and emotional states.

*Description:*

The environment of the waiting room was an important aspect of the waiting experience for many patients as it helped determine how calm or anxious the patients felt. Cleanliness was one factor that patients used to assess a waiting room. For many, it was the only factor they considered; if the room was clean, they were satisfied. As AQ put it: “As long as it’s clean here, I’m fine with it.” Some patients associated cleanliness with the people occupying the same space. As AH elucidated: “[the waiting room is] not clean, there are so many types of people”; and as BM put it (referring to another patient), “when that man stands up, his seat will be wet”. Since many patients prioritized their health and often saw their fellow patients as “dirty, filthy” (BL), personal space was highly valued. Patients preferred larger waiting rooms so that they could have more space and have more choices on where to sit.

Patients also wanted a waiting experience that was calming, with minimal noise. Many patients, such as BJ and AN, noted that the TV audio was bothersome and would prefer it to be off.

Patients also preferred waiting experiences that had distracting activities to calm them down. Suggestions included adding refreshments (coffee bars, water fountain, and snack machine) and things to do (magazines, computer, things for kids to play with, video games/entertainment), followed by improving the existing environment (making the chairs more comfortable, updating the pictures on the walls). When asked what benefit these would serve, they responded that such interventions would “help [them] calm down” (BL), “not stuff that’s rousing of emotion” (AN).

## Discussion

Our study used a mixed methods approach to understand the waiting experience for a medically underserved population. Quantitative analysis found that 74% of their time was spent waiting to see a provider or staff while in the waiting room or in the exam room. This corresponded with previous research that demonstrated that patients who were uninsured or had Medicaid experienced long wait times in comparison to the time spent with their provider [8, 9]. Qualitative analysis revealed that patient frustrations were rooted in the pre-visit experience and were further exacerbated during their visit. Insights 1–3 (pre-visit) illuminate the need for improved approaches to scheduling and access, as patients in medically underserved populations face unique barriers and opportunity costs in order to attend their appointments. Insights 4–6 (during visit), coupled with our quantitative findings of patient time tracking in clinic, demonstrated a poor patient experience exacerbated by information opacity, long wait times and an uncomfortable physical environment.

Using a mixed methods approach allowed us to use qualitative data to augment, add meaning to, and confirm findings from the quantitative data. For instance, our quantitative findings showed that patients spend the majority of their time waiting to be seen; our qualitative findings demonstrated that patients perceived this time as sources of anxiety and frustration and wondered whether the cost of waiting was justified. Most notably, qualitative analysis revealed that the ‘pre-visit’ waiting experience significantly impacts perceived satisfaction. Interventions based on quantitative data alone would have aimed at decreasing wait times and improving the experience of waiting in clinic, and thus would have fall short of meeting patients’ core needs. By using the lean methodology to assess the extent to which patients wait, and HCD to capture an in-depth view of patient frustrations and unmet needs, we can design patient-centered solutions that improve efficiency and experience.

This study had limitations. First, all interviews were conducted in one surgical waiting room at one hospital with a medically underserved population. As such, our results have limited generalizability for patients who might seek care at private hospitals. Second, interviews were conducted in the waiting room, which is a public space, rather than a private room, so that we could capture as many perspectives as possible. This meant that other patients could hear the conversation, which may have led to some response bias. For instance, while patients expressed anxiety that they would get sicker if they came into contact with other patients, many were hesitant to elaborate more about these fears within earshot of their fellow patients. Third, intercept interviews, by their nature, use convenient sampling. Eligible patients were those who were in the waiting room at the same time as when the researchers were conducting interviews. Also, given the nature of our study design, patient-specific sociodemographic information on the patients we observed and interviewed are not available. These may limit generalizability and internal validity, due to the possibility of sampling error and lack of representation of certain populations. As such, the exact insights and opportunities generated from this research are not directly generalizable to other contexts. However, the HCD and time tracking methodologies themselves are repeatable.

The surgical clinic where our study took place is at a safety-net hospital that treats patients who have a historically and/or personally poor relationship with the health system. A striking finding was that when interviewed, many patients felt hesitant to “complain” about their problems (AG, BE, BH, BL). Rather, they felt that their role was to be “patient” (AJ, AK, BH) and wait quietly for their turn. For instance, for AJ, waiting several months to be seen was the standard of healthcare delivery that he had experienced and so understood to be the norm: “There’s a wait time, sometimes 10-12 weeks, I understand, you just have to be patient, that’s what you expect at a hospital”. Another patient BH had already waited about 1 hour by the time we interviewed her. She was getting impatient since she had four young children under 10 years of age waiting at home, but didn’t think it was appropriate to ask to be seen. BH and others saw complaining as a character flaw; i.e., giving constructive feedback (something positive) had been internalized into something negative (complaining). By interpreting qualitative interviews through this lens, and realizing that some areas for improvement may go unrecognized and that others may be delivered in muted language and tone by our patients, we can help amplify their concerns and improve the care that they receive.

Our study must be considered within the broader context of racism and the inequities that it has brought and continues to bring into healthcare, especially in the U.S., and how those inequities impact our patients before they even enter the waiting room. Racism is a system of policies, practices, and norms that affects how people interact with the world based on their outer appearance. Historic policies, such as redlining, disproportionately affected and continue to affect people of color, placing them at higher risk for being poor, falling sick, and dying [34–36]. Such systems influence a person’s social circumstances, which are estimated to contribute to 24% of a person’s health status; medical care and the environment (under which the waiting experience falls) contributes to 18% [37–42]. At the local level, neighborhoods in San Francisco County with high rates of poverty are disproportionately composed of communities of color, have a higher density of stores that sell alcohol, tobacco, and fast foods, and a lower density of stores that sell fresh produce, lack of parks and open space, have limited public transportation, and have multiple sources of toxic exposures. These increase the risk for acute and chronic medical conditions such as heart disease, cancer, stroke, substance abuse, asthma, etc. [43] Our study contributes to the growing recognition and acknowledgement that the environments and processes that deliver healthcare to disadvantaged communities negatively impact their mental, emotional, and physical health.

## Conclusion

By using a mixed methods approach consisting of HCD in combination with lean methodology, we gained an in-depth understanding of the waiting experience in a general surgery outpatient clinic from the patient and system’s perspective. This dual approach, which places the patient at the center, will contribute to the development and implementation of patient-focused interventions that prioritize patients’ unmet needs.

## Data Availability

The datasets used and/or analysed during the current study are available from the corresponding author on reasonable request.
